# Imported human brucellosis in Belgium: Bio and molecular typing of bacterial isolates, 1996-2015

**DOI:** 10.1371/journal.pone.0174756

**Published:** 2017-04-06

**Authors:** Delphine Hanot Mambres, Samira Boarbi, Patrick Michel, Nora Bouker, Luisa Escobar-Calle, Damien Desqueper, Tiziano Fancello, Marjan Van Esbroeck, Jacques Godfroid, David Fretin, Marcella Mori

**Affiliations:** 1 Bacterial Zoonoses of Livestock, Operational Directorate Bacterial Diseases, Veterinary and Agrochemical Research Centre, CODA-CERVA, Brussels, Belgium; 2 National Reference Centre for Human Brucellosis, Brussels, Belgium; 3 Department of Clinical Sciences, Institute of Tropical Medicine, Antwerp, Belgium; 4 Faculty of Biosciences, Fisheries and Economics, University of Tromsø - The Arctic University of Norway, Tromsø, Norway; East Carolina University Brody School of Medicine, UNITED STATES

## Abstract

**Objectives:**

The aim of this study was to characterize by classical biotyping and Multi-Locus variable number tandem repeats (VNTR) Analysis (MLVA) all *Brucella spp*. derived from human cases in Belgium from 1996 to 2015. Final goals were to determine the species and biovar, to trace-back on genetic grounds the origin of each strain when patient history and risk factors were missing, and to survey for particular trends at the national level.

**Methods:**

A total of 37 *Brucella* strains, isolated from 37 patients in Belgium, were analyzed by both classical biotyping and MLVA, and the genetic patterns compared to those of human strains isolated worldwide.

**Results:**

Classical biotyping revealed that isolates were mainly *Brucella melitensis*. Most of them belonged to biovar 3, the most abundant biovar in the Mediterranean region. MLVA confirmed that *Brucella melitensis* is too diverse in VNTRs to be able to make clusters associated to each biovar, but it allowed retrieving precious epidemiological information. The analysis highlighted the imported nature of the strains from all over the world with a dominant part from the Mediterranean countries. Findings of the MLVA11 testing were in line with the travel history of patients coming from Italy, Turkey, Lebanon and Peru. The analysis was particularly useful because it suggested the geographical origin of the infection for 12/16 patients for whom no case history was available.

**Conclusion:**

Classical biotyping and MLVA analysis are not exclusive but remain complementary tools for *Brucella melitensis* strain surveillance. MLVA11 is sufficient for *Brucella*-free countries such as Belgium to trace the geographical origin of infection, but complete MLVA16 is needed to search for links with endemic areas.

## Introduction

Brucellosis is a bacterial zoonosis of economic and public health impact in many countries, especially in the Mediterranean area. It is caused by *Brucella spp*. and affects both domestic and wild animal species, besides humans. In animals, symptoms comprise abortion, infertility and reduction of milk production [1]. In humans, brucellosis manifests as a febrile, debilitating illness that, untreated, might evolve into chronic conditions (hepatomegaly, endocarditis, spondylitis, orchitis, arthritis, neurobrucellosis…) [2]. Currently, twelves species of *Brucella* have been identified [3], three causing proved zoonotic and economic threats: *B*. *melitensis*, *B*. *abortus* and *B*. *suis* which preferentially infect small ruminants, cattle and swine, respectively [4]. Classically, these species are subdivided in biovars: *B*. *melitensis* includes three biovars (1, 2 and 3), *B*. *abortus* eight (1, 2, 3, 4, 5, 6, 7 and 9), and *B*. *suis* five (1–5) [5][6]. The European Union has declared Belgium as Officially Free of *Brucella melitensis* (ObmF) in 1991 (Directive 91/68/EEC), and Officially Free of Bovine Brucellosis (OBF) (Commission Decision 2003/467/EC) in 2003. Various monitoring programmes, including serologic surveys in sheep and diagnosis of cattle abortions, are implemented to help preserve this status. Through these measures, early detection of *B*. *abortus* outbreaks in cows was achieved in 2010 and 2012 [7]. *Brucella melitensis* has never been isolated from animals in Belgium. However, other *Brucella spp*. circulate in the country: wild boars host *B*. *suis* biovar 2 [8] and marine mammals strains have been isolated on animals stranded on the Belgian coastline (unpublished data).

Human brucellosis is a mandatory notifiable disease in Belgium. Despite the *Brucella*-free status and the continuous monitoring, human brucellosis cases are reported yearly [9]. Applied case definition for human brucellosis is that recommended by the European Centre for Disease Prevention and Control [10]. Briefly, a confirmed case will meet both clinical (fever and one other generic symptoms of brucellosis) and laboratory criteria (isolation of the strain from clinical specimens and/or presence of *Brucella* specific antibodies in serum detected by agglutination tests or ELISA). In case of isolation, the identification of the bacterial species and its biovar is essential for epidemiological follow-up and control of the disease. Classical biotyping is the gold standard to investigate phenotypic characteristics [11]; this method becomes uncertain in case of appearance of mutants within the same species or biovar or the presence of strains with atypical reactions to microbiological tests [12]. Molecular genotyping methods assessing polymorphisms in the *omp2A* and *omp2b* genes [13] or by the sequencing of *rpoB* for example are helpful to confirm the species and the biovars of *Brucella* [14]. It is known that some human cases can emerge in ObmF countries with the source of contamination retrieved in infected endemic wildlife [15]. In this context, deeper molecular epidemiological tools become particularly interesting to trace-back the infection to identify the geographical source. To fulfill the objective of sub-species discrimination between strains, Variable Number Tandem Repeats (VNTR) has been investigated in Multi-Locus VNTR Analysis (MLVA) by various scientific groups since 2003 [6][16][17]. This method has proved its efficacy to obtain epidemiological information on *Brucella* strains, access to public databases and sharing of information between countries. In this study, MLVA was used to characterize Belgian human isolates collected during the national reference activity from 1996 to 2015.

## Methods

### Bacterial isolates

A total of 37 *Brucella* strains were isolated from 37 patients in Belgium between 1996 and 2015. They represent all human *Brucella* isolates collected during this period in Belgium. These strains were isolated from blood or cerebrospinal fluid at the first line laboratory or at the National Reference Centre for Brucellosis, Veterinary and Agrochemical Research Centre (CODA-CERVA) in Brussels. *Brucella melitensis* 16M was used as reference strain to calibrate the VNTR units.

### Biotyping

All isolates were identified as *Brucella* species on the basis of morphology and conventional microbiological procedures according to the OIE manual [18]: requirement of CO_2_ and O_2_ for growth, urease activity, H_2_S production, sensitivity to thionin (10 and 20 μg/ml), fuchsin (20 μg/ml) and saphranin (100 μg/ml) dyes and agglutination with monospecific antiserum for A and M antigens. Brucella monospecific antisera A and M were obtained from FAO/WHO Collaborating Centre for Brucellosis Reference and Research at the Veterinary Laboratory Agency, Weybridge, UK.

### DNA preparation, PCR and MLVA genotyping

A loopful of cultured bacterial cells were dissolved in water, heat treated at 99°C for 15 min and, after centrifugation, the supernatant was used as DNA template. PCR amplification was performed in a total volume of 25 μL containing 20 ng of DNA, 1x PCR reaction Buffer (Invitrogen), bethain 5M (Sigma), 1U of Taq DNA polymerase rec (Invitrogen), 50 mM of MgCl_2_, 5 mM of each dNTPs and 10 μM of each MLVA locus flanking primers [6]. The following PCR program with the thermocycler iCycler BioRad was used: an initial denaturating step at 96°C during 5 min followed by 30 cycles of 96°C for 30 sec, 60°C for 30 sec, 70°C for 1 min and a final extension step of 70°C for 5 min.

Genotyping was performed using a combination of both minisatellites and microsatellites repeats based on the scheme initially described by Le Fleche et al. [6], and adjusted by Al Dahouk et al. [17]. The tandem-repeat loci were divided into three groups as previously described [17]: eight minisatellite loci in panel 1 (bruce06, bruce08, bruce11, bruce12, bruce42, bruce43, bruce45, and bruce55), three microsatellite loci in panel 2A (bruce18, bruce19, and bruce21) (altogether MLVA11), and five microsatellite loci in panel 2B (bruce04, bruce07, bruce09, bruce16, and bruce30) (MLVA16).

For the markers bruce06, bruce11, bruce42, and bruce55 with repeat unit size of 134 bp, 63 bp, 125 bp and 40bp respectively, the PCR fragment size was evaluated by 2% agarose gel electrophoresis. DNA from the reference strain *B*. *melitensis* 16 M, for which repeats lengths are known, was used for standardization. A 100-bp ladder (Invitrogen) was used as molecular size marker. Ethidium bromide-stained gels were visualized by UV light and photographed with GeneGenius bio-imaging system (Syngene). For the markers bruce08, bruce12, bruce43, bruce45, bruce18, bruce19, bruce21, bruce04, bruce07, bruce09, bruce16 and bruce30, the PCR products length were defined by capillary electrophoresis with the CEQ 8000 Genetic Analysis System (Beckman Coulter, Indianapolis, IN, USA). The size of each PCR product was then converted to a corresponding tandem repeat number as described by Le Fleche et al. [6].

### Analysis of MLVA data

All data were analyzed using BioNumerics version 6.6 software (Applied Maths, Belgium). Clustering analysis was performed using categorical coefficient and the unweighted-pair group method with arithmetic mean algorithm (UPGMA) as indicated previously [17]. Briefly, three distinct character data sets with different weight were defined according to the diversity index of the markers and combined using the composite data set tool provided by Bionumerics. The first one corresponded to panel 1 markers. Each marker of this panel got an individual weight of 2 (total weight for panel 1: 16). The two others form two groups in the panel 2, 2A and 2B. Panel 2A markers got a weight of 1 (total weight for panel 2A: 3) and panel 2B markers got a weight of 0,2 (total weight of panel 2B: 1). The MLVA profile of the isolates was also subjected to a minimum spanning tree (MST) analysis, illustrating the diversity existing within the clusters based on single locus variations (SLV). Units (and not sizes) of each marker were considered for the analysis. The Hunter-Gaston Discrimination Index (HGDI) was calculated for each locus by the use of the online tool V-DICE (http://www.hpa-bioinformatics.org.uk/cgi-bin/DICI/DICI.pl, latest access February 25, 2017).

## Results

A total of 37 *Brucella* strains with an average of roughly two strains annually were isolated in Belgium during the reference activity between 1996 and 2015. Colony morphology, staining, growth characteristics, and slide agglutination with monospecific anti-*Brucella* sera were used to characterize all isolates (Table 1). These standard bacteriological procedures classified the bacterium at the species and the biovar levels. Globally, biotyping identified 28 strains as *B*. *melitensis* biovar 3, three strains as *B*. *melitensis* biovar 1, three as *B*. *melitensis* biovar 2, one as *B*. *melitensis* rough, and two as *B*. *abortus* biovar 1. The rough strain could not be typed (strain 16) and two smooth *B*. *melitensis* biovar 3 strains showed an atypical susceptibility to dyes and a delayed agglutination with anti-M serum (strain 32 and 37). Susceptibility to phages was tested in the latter strains which confirmed the identification of atypical *B*. *melitensis* biovar 3 strains.

**Table 1:** Results of the Belgian human *Brucella* strains—classical biotyping.

**Strain**	**CODA ID**	Year*	Land of origin**	**Species Biovar**	**Growth**	**CO2 need**	**H2S**	**O2**	**Urease**	**Anti A**	**Anti M**	**Thionin 10**	**Thionin 20**	**Fucsin 20**	**Saphranin 100**	**Tb RTD**	**Tb RTD 10^4**	**wb RTD**	**IZ RTD**
1	L3/09	1996	Morocco	B. melitensis bv 1	+	-	-	+	+	-	+	+	+	+	/	NA	NA	NA	NA
2	L3/10	1997	Turkey	B. melitensis bv 3	+	-	-	+	+	+	+	+	+	+	/	NA	NA	NA	NA
3	L3/14	1997	Turkey	B. melitensis bv 3	+	-	-	+	+	+	+	+	+	+	/	NA	NA	NA	NA
4	L3/15	1997	Italy	B. melitensis bv 3	+	-	-	+	+	+	+	+	+	+	+	NA	NA	NA	NA
5	L3/23	1998	?	B. melitensis bv 3	+	-	-	+	+	+	+	+	+	+	+	NA	NA	NA	NA
6	L3/107	1999	Peru	B. melitensis bv 1	+	-	-	+	+	-	+	+	+	+	+	NA	NA	NA	NA
7	L3/129	2000	?	B. abortus bv 1	+	+	+	-	+	+	-	-	-	+	+	NA	NA	NA	NA
8	L3/130	2001	?	B. melitensis bv 3	+	-	-	+	+	+	+	+	+	+	+	NA	NA	NA	NA
9	L3/131	2002	?	B. melitensis bv 3	+	-	-	+	+	+	+	+	+	+	+	NA	NA	NA	NA
10	L3/134	2002	?	B. melitensis bv 3	+	-	-	+	+	+	+	+	+	+	+	NA	NA	NA	NA
11	L3/135	2004	Libanon	B. melitensis bv 3	+	-	-	+	+	+	+	+	+	+	+	NA	NA	NA	NA
12	L3/136	2004	Libanon	B. melitensis bv 3	+	-	-	+	+	+	+	+	+	+	+	NA	NA	NA	NA
13	L3/137	2004	?	B. melitensis bv 3	+	-	-	+	+	+	+	+	+	+	+	NA	NA	NA	NA
14	L3/138	2004	?	B. melitensis bv 3	+	-	-	+	+	+	+	+	+	+	+	NA	NA	NA	NA
15	L3/139	2004	?	B. melitensis bv 3	+	-	-	+	+	+	+					NA	NA	NA	NA
16	L3/140	2004	?	B. melitensis rough	+	-	-	+	+	-	-	+	+	+	+	NA	NA	NA	NA
17	L3/141	2004	?	B. melitensis bv 3	+	-	-	+	+	+	+	+	+	+	+	NA	NA	NA	NA
18	L3/147	2005	?	B. melitensis bv 3	+	-	-	+	+	+	+	+	+	+	+	NA	NA	NA	NA
19	L3/148	2007	?	B. melitensis bv 2	+	-	-	+	+	+	-	+	+	+	+	NA	NA	NA	NA
20	L3/149	2007	?	B. melitensis bv 3	+	-	-	+	+	+	+	+	+	+	+	NA	NA	NA	NA
21	L3/150	2007	?	B. melitensis bv 2	+	-	-	+	+	+	-	+	+	+	+	NA	NA	NA	NA
22	L3/173	2010	Ecuador	B. abortus bv 1	+	+	+	-	+	+	-	-	-	+	+	NA	NA	NA	NA
23	L3/200	2011	China	B. melitensis bv 3	+	-	-	+	+	+	+	+	+	+	+	NA	NA	NA	NA
24	L3/201	2011	Turkey	B. melitensis bv 3	+	-	-	+	+	+	+	+	+	+	+	NA	NA	NA	NA
25	L3/202	2011	Turkey	B. melitensis bv 2	+	-	-	+	+	+	-	+	+	+	+	NA	NA	NA	NA
26	L3/203	2011	Turkey	B. melitensis bv 3	+	-	-	+	+	+	+	+	+	+	+	NA	NA	NA	NA
27	L3/204	2011	Turkey	B. melitensis bv 3	+	-	-	+	+	+	+	+	+	+	+	NA	NA	NA	NA
28	L3/205	2012	Turkey	B. melitensis bv 3	+	-	-	+	+	+	+	+	+	+	+	NA	NA	NA	NA
29	L3/206	2012	Turkey	B. melitensis bv 3	+	-	-	+	+	+	+	+	+	+	+	NA	NA	NA	NA
30	L3/251	2012	Spain	B. melitensis bv 3	+	-	-	+	+	+	+	+	+	+	+	NA	NA	NA	NA
31	L3/278	2014	?	B. melitensis bv 3	+	-	-	+	+	+	+/-	+	-	-	-	-	-	-	+-
32	L3/289	2015	?	B. melitensis bv 3	+	-	-	+	+	+	+/-	+	+	+/-	-	NA	NA	NA	NA
33	L3/295	2015	Italy	B. melitensis bv 3	+	-	-	+	+	+	+	+	+	+	+	-	-	-	+-
34	L3/296	2015	Italy	B. melitensis bv 3	+	-	-	+	+	+	+	+	+	+	+	-	-	-	+-
35	L3/297	2015	Italy	B. melitensis bv 3	+	-	-	+	+	+	+	+	+	+	+	-	-	-	+-
36	L3/298	2015	Afghanistan	B. melitensis bv 1	+	-	-	+	+	-	+	+	+	+	+	NA	NA	NA	NA
37	L3/311	2015	Turkey	B. melitensis bv 3	+	-	+/-	+	+	+	+/-	+	+	+/-	-	-	-	-	+-

*Year of isolation;

** Land of origin is based on the travel history of the patient.

Highlighted in light grey are atypical reactions.

NA = Not Applicable

All isolates were further characterized by MLVA (Table 2). The 16 VNTRs were split into three panels (1, 2A and 2B) [17] because of the difference in their diversity index. Three MLVA analyses were run depending on the panels considered (MLVA8 = panel 1, MLVA11 = panel 1 + panel 2A, MLVA16 = panel 1 + panel 2A + panel 2B). A comparison of the Belgian isolates with the strains present in the public repository by MLVA8 and MLVA11 [19] allowed the identification of the associated genotypes (Table 2). Fourteen genotypes were identified with respect to the MLVA8; five were new and received numbering as “184- to 188”. These genotypes were all single-locus variants of known genotypes. Nineteen genotypes were identified with MLVA11; eight as new, numbered “361- to 368”, seven as single-locus variants and one as a two-locus variant.

**Table 2:** MLVA16 genotype of the Belgian human *Brucella* strains.

				Panel1								Panel2A			Panel2B				
Strain	CODA ID	MLVA 8	MLVA11	Bruce06	Bruce08	Bruce11	Bruce12	Bruce42	Bruce43	Bruce45	Bruce55	Bruce18	Bruce19	Bruce21	Bruce04	Bruce07	Bruce09	Bruce16	Bruce30
1	L3/09	140	361	2	5	3	13	4	2	3	5	5	36	8	4	5	7	8	5
2	L3/10	43	104	1	5	3	13	3	2	3	2	5	36	8	5	4	3	8	6
3	L3/14	42	116	1	5	3	13	2	2	3	2	4	41	8	5	7	3	5	5
4	L3/15	51	91	3	5	3	13	1	1	3	3	8	43	8	6	8	8	5	3
5	L3/23	184	362	1	5	3	13	2	2	3	3	4	41	8	4	11	3	7	5
6	L3/107	80	133	3	4	2	13	3	2	3	3	7	36	6	2	4	8	4	4
7	L3/129	28	82	4	5	4	12	2	2	3	3	6	43	8	3	1	6	3	6
8	L3/130	43	125	1	5	3	13	3	2	3	2	4	41	8	5	4	7	4	3
9	L3/131	42	116	1	5	3	13	2	2	3	2	4	41	8	5	6	3	6	6
10	L3/134	43	125	1	5	3	13	3	2	3	2	4	41	8	5	4	3	6	5
11	L3/135	44	110	1	4	3	13	3	2	3	2	4	41	8	8	4	3	7	5
12	L3/136	44	110	1	4	3	13	3	2	3	2	4	41	8	8	4	3	7	5
13	L3/137	42	116	1	5	3	13	2	2	3	2	4	41	8	5	7	3	8	5
14	L3/138	42	116	1	5	3	13	2	2	3	2	4	41	8	6	10	3	6	5
15	L3/139	45	115	1	5	3	12	2	2	3	2	4	41	8	4	5	3	6	7
16	L3/140	51	96	3	5	3	13	1	1	3	3	7	43	8	5	6	10	5	3
17	L3/141	43	125	1	5	3	13	3	2	3	2	4	41	8	5	4	3	10	5
18	L3/147	49	87	3	6	3	14	1	1	3	3	7	43	8	5	7	12	6	3
19	L3/148	43	125	1	5	3	13	3	2	3	2	4	41	8	4	4	3	9	5
20	L3/149	43	125	1	5	3	13	3	2	3	2	4	41	8	4	4	3	5	4
21	L3/150	43	125	1	5	3	13	3	2	3	2	4	41	8	6	4	3	3	4
22	L3/173	185	363	4	6	3	12	2	2	3	3	6	43	8	5	4	3	5	5
23	L3/200	83	298	1	5	3	14	2	2	3	2	4	41	8	6	5	3	9	9
24	L3/201	42	116	1	5	3	13	2	2	3	2	4	41	8	4	4	3	5	4
25	L3/202	43	125	1	5	3	13	3	2	3	2	4	41	8	6	4	3	7	4
26	L3/203	43	125	1	5	3	13	3	2	3	2	4	41	8	5	5	3	3	4
27	L3/204	43	125	1	5	3	13	3	2	3	2	4	41	8	5	5	3	3	4
28	L3/205	186	364	1	3	3	13	3	2	3	3	4	41	8	5	5	3	3	4
29	L3/206	187	365	1	5	3	13	3	2	3	3	4	41	8	5	4	3	4	4
30	L3/251	188	366	3	5	3	13	2	1	3	3	7	43	8	5	4	9	10	3
31	L3/278	42	367	1	5	3	13	2	2	3	2	4	42	8	8	6	11	7	6
32	L3/289	42	116	1	5	3	13	2	2	3	2	4	41	8	4	7	3	7	6
33	L3/295	51	96	3	5	3	13	1	1	3	3	7	43	8	6	3	11	5	3
34	L3/296	51	96	3	5	3	13	1	1	3	3	7	43	8	6	3	11	5	3
35	L3/297	51	96	3	5	3	13	1	1	3	3	7	43	8	6	3	11	5	3
36	L3/298	42	116	1	5	3	13	2	2	3	2	4	41	8	3	4	3	4	5
37	L3/311	42	368	1	5	3	13	2	2	3	2	4	40	8	9	7	3	7	5

MLVA8 and MLVA11 genotypes were derived by comparison with data in http://microbesgenotyping.i2bc.paris-saclay.fr/. Each marker was calibrated by the 16M *B*. *melitensis* reference strain units.

**Fig 1:**
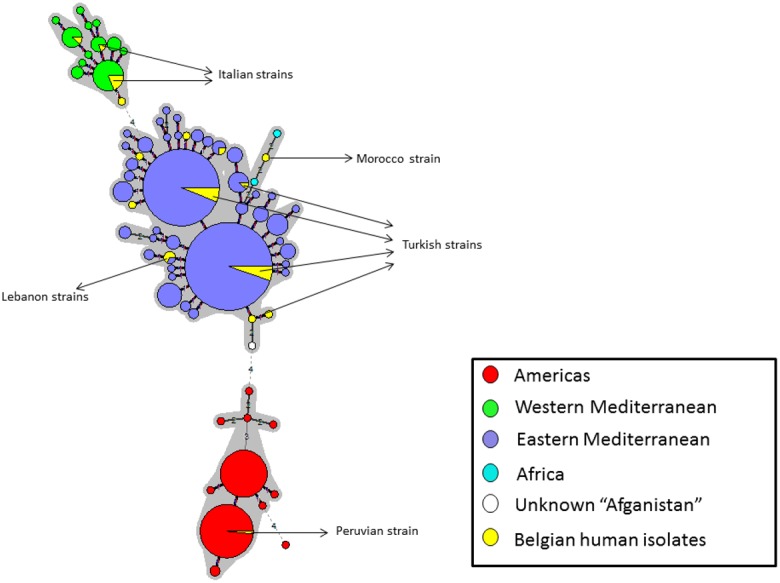
Minimum spanning tree obtained with MLVA-11 data of Belgian human isolates together with human strains worldwide (public databases). Each strain is represented by a circle. Large circles regroup strain with identical genotypes dimensionally proportional to the number of entries. The lines, whose length is drawn on the basis of the number of mutations (number of variant VNTR), connect the different genotypes. The numbers aside the lines indicate the number of variant VNTRs. The grey areas connect genotypes with maximum three-locus variants. The East Mediterranean group is colored in blue, the America group in red, the West Mediterranean group in green, and the Belgian strains in yellow. Arrows describe origins for some Belgian isolates.

MLVA11 data were used to draw a minimum spanning tree (MST) of *Brucella melitensis* strains comprising the human Belgian isolates and the human strains isolated worldwide [19] (Fig 1). Three main clusters were derived, corresponding to the origin of the isolates: an East Mediterranean, a West Mediterranean and an American cluster. Belgian human strains did not cluster within a homogenous cloud, indicating the diversity and suggesting that these isolates were imported. A more detailed link with the geographical origin of some Belgian isolates could be established (Fig 1 and Table 3). The strains isolated from patients infected in Italy clustered separately from the other strains, and demonstrated genotypes described for Italian strains (genotype 51 for MLVA8 and genotypes 91 and 96 for MLVA11). The strains from patients with a history of contamination in Turkey had genotypes comparable to those described for Turkish strains in the database (mainly genotypes 42 and 43 for MLVA8 and genotypes 104, 116 and 125 for MLVA11). The only strain isolated from a patient traveling from Peru felt within the Americas cluster and showed the same genotype as Peruvian strains present in the public database. The strain of a patient traveling from Morocco had a genotype close to an African strain in the database. MLVA8 and 11 results suggested a possible origin of the infection for 12 out of 16 Belgian strains for which information about the patient or stay abroad was unknown (Table 3). For example, strains 8, 9, 10, 13, 14, 17, 19, 20, 21 and 32 had typical genotypes of Turkish strains (42 and 43 for MLVA8 and 116 and 125 for MLVA11), strain 16 demonstrated the same genotype as Italian strains (51 for MLVA8 and 96 for MLVA11) and strain 15 had a typical genotype of Chinese strains (45 for MLVA8 and 115 for MLVA11). Little information can be drawn regarding the *Brucella abortus* strains isolated in Belgium. One of them had the same genotypes (28 for MLVA8 and 82 for MLVA11) as hundreds of strains isolated all over the world, mainly from cattle. The other strain had a new genotype.

**Table 3:** MLVA11 predicted geographical origin of the Belgian human *Brucella* strains.

Strain	CODA ID	Land of origin by information	Predicted land of origin by MLVA 11
1	L3/09	Morocco	Morocco
2	L3/10	Turkey	Turkey
3	L3/14	Turkey	Turkey
4	L3/15	Italy	Italy
5	L3/23	?	?
6	L3/107	Peru	Peru
7	L3/129	?	?
8	L3/130	?	Turkey
9	L3/131	?	Turkey
10	L3/134	?	Turkey
11	L3/135	Libanon	Libanon
12	L3/136	Libanon	Libanon
13	L3/137	?	Turkey
14	L3/138	?	Turkey
15	L3/139	?	China
16	L3/140	?	Italy
17	L3/141	?	Turkey
18	L3/147	?	?
19	L3/148	?	Turkey
20	L3/149	?	Turkey
21	L3/150	?	Turkey
22	L3/173	Ecuador	Ecuador
23	L3/200	China	China
24	L3/201	Turkey	Turkey
25	L3/202	Turkey	Turkey
26	L3/203	Turkey	Turkey
27	L3/204	Turkey	Turkey
28	L3/205	Turkey	Turkey
29	L3/206	Turkey	Turkey
30	L3/251	Spain	Spain
31	L3/278	?	?
32	L3/289	?	Turkey
33	L3/295	Italy	Italy
34	L3/296	Italy	Italy
35	L3/297	Italy	Italy
36	L3/298	Afghanistan	Afghanistan
37	L3/311	Turkey	Turkey

The complete MLVA16 was used to generate a dendrogram (S1 Fig) revealing that the 37 Belgian isolates clustered in 33 different genotypes. This analysis confirmed that the isolates came from multiple geographical origins but did not provide additional epidemiological information.

In certain contexts, MLVA16 can be used to cluster the strains regarding to the biovars, [6] [20]. For *B*. *melitensis* this seems to be not effective [17] [21]. Despite the use of three levels of analysis (MLVA8/MLVA11/MLVA16), it was not possible to cluster the Belgian *B*. *melitensis* strains according to their biovar. In some cases the three described biovars (bv1; bv2 and bv3) were found within the same cluster (S1 Fig).

The HDGI for the different MLVA16 loci were calculated and confirmed the high discriminatory power of MLVA (S1 Table).

## Discussion

Brucellosis is a worldwide zoonotic disease that affects various domestic and wild animals but some *Brucella* species are occasionally able to infect humans, considered as accidental hosts. The transmission occurs by aerosol, consumption of raw dairy products or direct contact with contaminated animals. Human brucellosis is characterized by a febrile debilitating disease that can become a chronic infection accompanied with serious complications if not adequately treated. The impact of the infection on health, the mode of transmission and the zoonotic aspect of the disease have driven European countries to impose a compulsory declaration for each confirmed case to the reference centres. In Belgium, like in other OmbF and OBF countries, human brucellosis is limited to few cases every year and its incidence is low. The majority of the human cases diagnosed in Belgium are due to travel in endemic countries and to immigrations because of the absence of animal control measures in many parts of the world. Epidemiological surveillance in *Brucella*-free countries remains therefore essential. In Belgium, some *Brucella* species (*B*.*suis*, *B*. *ceti* and *B*. *pinnipedialis*) reside in wildlife and it was recently shown that clustered outbreaks in domestic animals have occurred. *Brucella* species are genetically close to each other [22] and, despite their host preference, some species are able to cross the host specificity barrier [23][24]. This zoonotic characteristic inevitably justifies passive surveillance of brucellosis in humans. Classical biotyping is the gold standard for species and biovar attribution of the isolates but the information lacks resolution. In some cases, appearance of mutants that generate genetic diversity is responsible for atypical susceptibility to dyes or delayed agglutination with anti-*Brucella* sera and thus hinders an accurate identification of the species and biovar [12]. Molecular genotyping based on single gene or multilocus sequence typing to identify locus polymorphisms or other mutations in genes have also been developed to understand the phylogeny of the genus *Brucella* and try to address the question of species and biovars [25][26]. These methods are not exhaustive when sub-typing for epidemiological trace-back is necessary. Since 2002, when the first complete genomic sequences of *Brucella melitensis* and *suis* [27][28] were released, a number of tools have been developed to differentiate the isolates according to DNA repetitive sequences. A first application of microsatellites fingerprinting to *Brucella* strains was made in 2003 [16] and was based on nine VNTRs that allowed discrimination of some isolates beyond the biovar level [29]. Afterwards, other sets of VNTR markers with a different speed of evolution were identified that demonstrated higher discriminatory power [6][30]. The group of Al Dahouk modified the set of VNTR markers of Le Fleche and showed their ability to cluster *Brucella melitensis* strains in 3 main geographical groups. In our study, the complete VNTR panel for *Brucella* was analyzed in strains isolated from humans in Belgium from 1996 to 2015 to improve their characterization and evaluate their origin. When compared with biotyping, we could assess the ability of VNTR in the identification of the biovars of *Brucella*.

Previous studies showed that MLVA was able to discriminate biovars of *Brucella* species such as *B*. *suis* and *B*. *abortus* [6]. However, it was also shown that the technique has its limitations with exceptions [31] and that it is not possible to discriminate biovars of the *B*. *melitensis* species [17]. Our study confirmed that the high genetic diversity regarding MLVA in this species did not allow clustering it according to the biovars. Therefore, classical biotyping and MLVA characterization remain complementary tools for *Brucella* strain surveillance. VNTRs were interesting to understand the epidemiology of the isolated strains. The study of MLVA11 was effective to establish phylogeographic relations of the Belgian strains with strains present in public databases. For instance, the single strain belonging to the Americas cluster was isolated in a patient that travelled to Peru. It shared the same genotype as 62 strains coming from Peru described by various groups [32][33]. Six isolates displayed the exact MLVA11 genotypes of dozens of strains coming from Italy described previously [6][12][17]. While the Italian origin for 5 of these Belgian isolates was known, thanks to the databases, it is suspected that the 6^th^ patient also contracted the disease in this country. These 6 strains belonged to the West Mediterranean cluster. A last isolate belonging to this cluster was a new genotype based on a single locus variant with Italian strains and was isolated from a patient with a travel history in Spain. The East Mediterranean cluster contained the majority of the strains isolated in Belgium. Nine of them shared the same MLVA11 genotype (genotype 43) with dozens of strains coming from Turkey as previously described [17] [34]. Few strains with the same MLVA11 genotype come from Syria and Irak [17]; this genotype was also observed in strains coming from Lebanon [35]. Seven other strains isolated in Belgium shared the same genotype with a great panel of strains isolated in China [21] and Turkey [17][34] (genotype 42). Information about the genotypes present in countries geographically located between Turkey and China is missing but this genotype 42 seems to be highly represented in the Middle East [17][35] and has already been isolated in countries like Kazakhstan [17][36]. We hypothesize that this particularly stable genotype [21] is largely spread from Turkey to China and that MLVA11 is not variable enough to distinguish strains from this region of the world. Our hypothesis is sustained by an MST analysis performed on 446 *B*. *melitensis* MLVA15 profiles of strains belonging to the East Mediterranean group [37]. In this study, part of the Turkish genotypes clustered with most of the genotypes observed in China and Kazakhstan, suggesting a common origin between strains from the Asian continent and the East Mediterranean area [37].

When examined with MLVA16 which includes panel 2B composed of five unstable satellites with a high mutation rate, Belgian isolates were found highly diverse; 33 genotypes were distinguished among the 37 isolated strains. They apparently came from all around the Mediterranean region except one strain with a genotype clustering together with strains from Peru. MLVA16 did not add to the information obtained already by MLVA11 that strains isolated in Belgium were coming from a large panel of areas in the world. It seems that increasing the number of markers is particularly useful in case of outbreak investigation in endemic countries. For highly genetic diverse strains such as in the context of imported cases, MLVA11 is in general sufficient to trace the geographical origin of infection. Accessibility of MLVA16 information in public repositories about human strains isolated in Belgium (and worldwide) could help endemic countries to better investigate a possible link and the spread with their cases.

Classical biotyping revealed that the human cases in Belgium are mainly *Brucella melitensis*, a species never isolated in animals in Belgium. This is in agreement with the fact that *B*. *melitensis* is much more common than *B*.*abortus* in many countries of Eastern Europe and the Middle East [38]. More precisely, most of the strains belonged to the biovar 3. This is consistent with the fact that the majority of patients travelled to or originated from Mediterranean countries and the Middle East where this biovar is predominant [11]. Since 1996 only two human isolates in Belgium revealed to be *B*. *abortus*, both belonging to biovar 1. One of the two patients was coming from Ecuador. To our knowledge, *B*. *abortus* is the only species isolated in humans in this country [39]. We do not have any information about the second patient but it is worthwhile to note that *Brucella abortus* biovar 1 was not enzootic in Belgium during the years 1980–2000. It has only been isolated in the late nineties from cattle imported from Portugal where this biovar was prevalent. Taken together, this information convincingly indicate that the majority of human brucellosis cases in Belgium is associated with travel to *Brucella* endemic countries as observed in neighbouring countries [40].

Our study is the first characterization of human *Brucella* isolates in Belgium. It highlights the genetic diversity of the strains consistent with the imported nature of the infections and the use of MLVA as an important tool to improve the understanding of the phylogeography of *Brucella*.

## Supporting information

S1 Fig Dendrogram using MLVA16 data. Yellow dots correspond to Belgian strains.
(TIF)

S1 Table Hunter Gaston diversity index for the different MLVA16 loci in Belgian *B*.*melitensis* strains. (DOCX)
