# Supplementary figures and images for: Imported human brucellosis in Belgium: Bio and molecular typing of bacterial isolates, 1996-2015
Source: PLoS One. 2017 Apr 6;12(4):e0174756. doi: 10.1371/journal.pone.0174756 (PMC5383062; doi:10.1371/journal.pone.0174756)

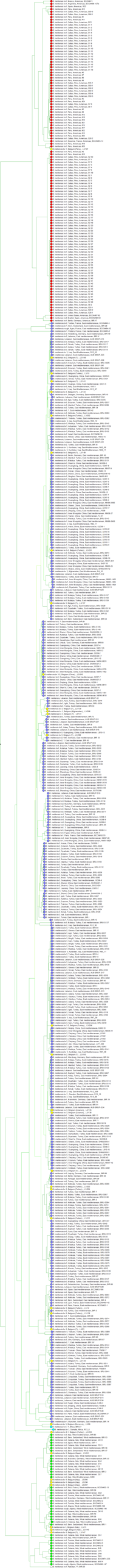

Supplement: S1 Fig — Yellow dots correspond to Belgian strains. (TIF) [file pone.0174756.s001.tif]
