# Supplementary material for: Imported human brucellosis in Belgium: Bio and molecular typing of bacterial isolates, 1996-2015
Source: PLoS One. 2017 Apr 6;12(4):e0174756. doi: 10.1371/journal.pone.0174756 (PMC5383062; doi:10.1371/journal.pone.0174756)
Supplement: S1 Table — (DOCX) [file pone.0174756.s002.docx]

**S1 Table:** Hunter Gaston Diversity Index for the different MLVA16 loci in Belgian *B.melitensis* strains.

| **Locus** | **Diversity Index** | **Confidence Interval** | **K** | **max(pi)** |
| --- | --- | --- | --- | --- |
| Bruce06 | 0.407 | 0.250 - 0.564 | 3 | 0.743 |
| Bruce08 | 0.264 | 0.080 - 0.448 | 4 | 0.857 |
| Bruce11 | 0.057 | 0.000 - 0.161 | 2 | 0.971 |
| Bruce12 | 0.165 | 0.004 - 0.325 | 3 | 0.914 |
| Bruce42 | 0.667 | 0.597 - 0.737 | 4 | 0.429 |
| Bruce43 | 0.329 | 0.170 - 0.488 | 2 | 0.800 |
| Bruce45 | 0.000 | 0.000 - 0.180 | 1 | 1.000 |
| Bruce55 | 0.482 | 0.363 - 0.602 | 3 | 0.657 |
| Bruce18 | 0.459 | 0.289 - 0.628 | 4 | 0.714 |
| Bruce19 | 0.534 | 0.370 - 0.698 | 5 | 0.657 |
| Bruce21 | 0.057 | 0.000 - 0.161 | 2 | 0.971 |
| Bruce04 | 0.760 | 0.676 - 0.843 | 7 | 0.400 |
| Bruce07 | 0.771 | 0.666 - 0.877 | 8 | 0.429 |
| Bruce09 | 0.523 | 0.333 - 0.712 | 7 | 0.686 |
| Bruce16 | 0.872 | 0.833 - 0.912 | 8 | 0.229 |
| Bruce30 | 0.771 | 0.712 - 0.831 | 6 | 0.343 |

**Notes** (from http://www.hpa-bioinformatics.org.uk/cgi-bin/DICI/DICI.pl)

Diversity Index (for VNTR data) = A measure of the variation of the number of repeats at each locus. Ranges from 0.0 (no diversity) to 1.0 (complete diversity)

Confidence Interval = Precision of the Diversity Index, expressed as 95% upper & lower boundaries

K = Number of different repeats present at this locus in this sample set

max(pi) = Fraction of samples that have the most frequent repeat number in this locus (range 0.0 to 1.0)
